# A Cross-Sectional Study on the Flood Emergency Preparedness among Healthcare Providers in Saudi Arabia

**DOI:** 10.3390/ijerph18031329

**Published:** 2021-02-02

**Authors:** Ahmed M. Al-Wathinani, Abdulaziz Alakeel, Ahmad Hecham Alani, Mohammad Alharbi, Abdullah Almutairi, Tahani Alonaizi, Riyadh A. Alhazmi, Sultan M. Alghadeer, Abdulmajeed M. Mobrad, Krzysztof Goniewicz, Amir Khorram-Manesh, Attila J. Hertelendy

**Affiliations:** 1 Department of Emergency Medical Services, Prince Sultan Bin Abdulaziz College of Emergency Medical Services, King Saud University, Riyadh 11451, Saudi Arabia; rialhazmi@ksu.edu.sa (R.A.A.); amobrad@ksu.edu.sa (A.M.M.); 2 National Pharmacovigilance and Drug Safety Center, Saudi Food and Drug Authority, Riyadh 33292, Saudi Arabia; Anaqeel@sfda.gov.sa; 3 Independent Researcher, London EC2V 7AN, UK; ahmad.hechamalani@rescue.org; 4 Public Health Department, Ministry of Health, 34496 Jeddah, Saudi Arabia; malharbi26@moh.gov.sa; 5 Infection Control Department, King Khalid Hospital, Ministry of Health, Riyadh 12372, Saudi Arabia; abnealmutairi@moh.gov.sa; 6 Department of Nursing, King Fahad Medical City, Ministry of Health, Riyadh 12231, Saudi Arabia; tnmalenizi@kfmc.med.sa; 7 Basic Science Department, Prince Sultan Bin Abdulaziz College of Emergency Medical Services, King Saud University, Riyadh 11451, Saudi Arabia; salghadeer@ksu.edu.sa; 8 Basic Science Department, Prince Sultan Bin Abdulaziz College of Pharmacy, King Saud University, Riyadh 11451, Saudi Arabia; 9 Department of Aviation Security, Military University of Aviation, 08521 Deblin, Poland; k.goniewicz@law.mil.pl; 10 Department of Surgery, Institute of Clinical Sciences, Sahlgrenska Academy, Gothenburg University, 41345 Gothenburg, Sweden; amir.khorram-manesh@surgery.gu.se; 11 Department of Development and Research, Armed Forces Center for Defense Medicine, 42676 Gothenburg, Sweden; 12 Department of Information Systems and Business Analytics, College of Business, Florida International University, Miami, FL 33174, USA; ahertele@fiu.edu

**Keywords:** flood, disasters, emergency preparedness, hospital preparedness, Saudi Arabia

## Abstract

This study used a descriptive cross-sectional methodology to measure healthcare workers’ knowledge, attitudes, perceptions, and willingness to respond to a flood scenario in Saudi Arabia. A validated survey was distributed to collect data using a convenience sampling technique through multiple social media platforms. A total of 227 participants were included in this study: 52% of them were aged between 26 to 34 years, 74% were residents from Riyadh, and 52.4% worked in nursing divisions. A significant number of respondents (73.2%) had positive perceptions towards their hospitals’ ability to provide an effective response to a flood, 89% were willing to report to work following a flood, and 90% of participants reported the need to develop both guidelines and training for flood disaster preparedness. Preparation and successful flood mitigation in the hospital setting requires staff that have both knowledge and training in emergency management. One way to obtain such readiness is through competency-based training, including both table-top and full-scale live exercises. Although the willingness to respond to such a flooding emergency was high among staff, the development of guidelines and educational programs is needed in order to develop the competencies and skills sets to improve disaster preparedness response and preparedness efforts.

## 1. Introduction

Morbidity and mortality attributed to flooding can either be caused directly by drowning, electrical shock injuries, and the transmission of communicable diseases, or indirectly by affecting infrastructure or other essential necessities of life and the interruption of fundamental public health services [1]. Floods can, in general, be categorized into either flash flood events or associated with cyclones, tsunamis, or storm surges [1]. As a result of global warming, climate change has also increased the risk of floods [2]. According to the Organization for Economic Cooperation and Development, the global cost of floods is approximately 40 billion USD per annum [3]. This type of natural hazard is projected to raise the global burden of disease, morbidity, mortality, and social and economic instability and place continued stress on healthcare systems [4].

Saudi Arabia (SA) is a disaster-prone country. Flooding, although infrequent, has posed significant challenges in the past [5]. The country has reported 14 floods that have impacted approximately 30,000 citizens and resulted in economic losses of about $450 million during the last three decades [5]. Examples include Makkah’s 2003 flood, which was the worst in the past 25 years [6]. Then in 2004, Jizan had experienced one of the most devastating floods in 45 years. Widespread devastation was reported during Medina’s flood in 2005. Jeddah’s floods occurred in 2009 and 2011, which resulted in 172 deaths [7]. In the capital city, Riyadh, floods in 2005 and 2010 resulted in numerous deaths and forced others to evacuate [6]. The frequency of flood occurrences in SA is expected to be at least seven times per year on average, mainly during the winter, and affecting all regions of the country [7,8,9]. Additionally, projected rainfall trends during 2025–2044, 2045–2064, and 2065–2084 based on data coming from the National Center for Atmospheric Research (NCAR) Community Climate System Model (CCSM4) have showed variable patterns, with significant increases in certain regions of SA [10]. It was reported that hospitals in Jeddah had faced a major crisis in 2009 as a result of the aforementioned floods. It raised many concerns at the Ministry of Health (MOH) and with other authorities about the preparedness of hospitals in SA [8].

The healthcare infrastructure in SA is managed by the MOH. Services are made available to the public through a network of 244 hospitals and 2037 primary healthcare centers across the country [6]. However, other governmental agencies also provide healthcare services independently of the MOH. These include the Ministry of Defense and Aviation (MODA), the Ministry of Education (MOE), the Saudi Arabian National Guard (SANG), the Ministry of the Interior (MOI), and the Red Crescent Society [6].

The Presidency of Meteorology and Environment is responsible for disaster risk reduction efforts in SA, and the Civil Defense at the MOI is responsible for emergency planning and response [6,9]. The current disaster risk management paradigm does not utilize a coordinated multi-agency level response approach. Instead, practice remains unconventional and subject to regional variations [11,12]. Alshehri and colleagues surveyed the public in Saudi Arabia, where a majority believed that God is in control of the world, and that disasters may be considered as punishment by God for transgressions by mankind [13]. The study recommended a focus on public awareness in terms of education, training, and volunteering in an effort to improve disaster readiness in the country [12]. Another study by Abosuliman et al. emphasized the need for the identification and coordination of organizational responsibilities and advocated for response team training [14]. Finally, a recent study by Al-Shareef et al. suggested that some hospitals might be inadequately prepared for future disasters in SA [15]. These studies show a significant shortcoming in response capabilities and civilian preparedness.

Floods are considered a major disaster in SA with the potential to disrupt the lives of residents, businesses, and critical government infrastructure, such as hospitals [6]. Flood-related disruptions may come in many different forms. For instance, Thailand’s 2011 flood resulted in many damaged hospitals, unavailability and/or disruption of supply chains, and staff shortages [16]. Several studies have reported an unwillingness of healthcare workers to return to work following a disaster, in addition to a general lack of knowledge related to disaster preparedness and response [17,18,19]. While there is a strong health system operating in SA, improvements are needed in localized and appropriate disaster-related training and investments in workforce education in order to strengthen flood resilience.

The aim of this study was to evaluate healthcare workers’ (HCWs) knowledge, attitudes, and perceptions of their preparedness and willingness to respond properly during future flood disasters in the Kingdom of Saudi Arabia.

## 2. Materials and Methods

### 2.1. Study Design

A descriptive, cross-sectional study design that measured HCWs’ willingness to respond, knowledge levels, attitudes, and perceptions with regard to a flood scenario at their hospital was utilized. Ethical approval was obtained from the Institutional Review Board at King Saud University Medical City. Informed consent was completed electronically. Only anonymous data were kept and shared with the study team. Participants who agreed to give their consent were included in the analysis. The study took place in SA between December 2019 and April 2020.

### 2.2. Variables

Independent variables included in this study were age, gender, marital status, number of residing children, type of occupation, years of service within the hospital, and the scope of hospital practice, (governmental vs. private). Dependent variables consisted of general knowledge and perceptions towards floods, willingness to report to work following a disaster, and knowledge concerning a flood scenario.

### 2.3. Sample Size

According to published data from the McKinsey Global Institute, the total number of males and females working in the healthcare sector in SA in 2014 was 600,000, with 350,000 healthcare professionals and 250,000 management and other support staff [20]. To facilitate the identification of differences and similarities, and to illustrate the complexity of this issue between the participants regarding each section of the survey, it was estimated that 196 participants were needed, while fixing the marginal error to 7% and a 95% confidence interval.

### 2.4. Enrolment

The study participants were HCWs of both genders who were working and living in SA. The process of enrolment was completed anonymously and voluntarily. Data was collected using a convenience sampling technique. To reach our target population, the survey was disseminated electronically using various social media platforms (WhatsApp, Telegram, Twitter, and Instagram) targeting groups and accounts known to be an aggregate of healthcare professionals where information around continuing medical education activities are shared [21]. The link to the survey was shared over a period of 14 days.

### 2.5. Data Collection Tools and Procedures

A recently developed and validated tool to model HCWs’ willingness to respond to an earthquake scenario was used in this study by changing the scenario to flooding. This survey initially aimed to measure HCWs’ willingness to respond to a variety of emergency situations. Previous studies have described in detail the design and validation process [16,17]. The flooding scenario was adopted as it is the country’s most common natural catastrophe, causing 7 out of 10 of the most devastating natural disasters in the history of SA between 1900 and 2010 [9], and because certain regions of the country are also projected to experience future trends of increased precipitation and extreme rainfall events [10].

The final version of the survey is composed of 34 items. The data collection tool is divided into two sections: one for the demographic information of participants, and the other measures knowledge and perceptions. The latter section contains questions related to HCWs and perceptions towards their roles following a flood, knowledge, competency, and willingness to report to work in the event of a flood scenario, and the factors that may influence their decisions in such circumstances. Lastly, participants were also asked about their perceptions in terms of guideline development and training sessions on flood disasters. The final questionnaire’s presentation, in terms of feasibility, readability, accuracy, design and formatting, and quality of the vocabulary used, was subjected to face validity checks with 10 volunteer experts from King Saud University Medical City. An Arabic version was available, which was translated by two authors and piloted on the same volunteers for validation. A scoring system was developed for knowledge questions: it considers zero to three correct answers as a low level of knowledge, four to seven correct answers as a moderate level of knowledge, and eight to twelve correct answers as a high level of knowledge.

### 2.6. Data Analysis

All data analyses were performed using International Business Machines (IBM, Armonk, NY, USA) Statistical Package for the Social Sciences (SPSS) 20.0 software (SPSS Inc., Chicago, IL, USA). Demographic data was analyzed and presented using frequencies and percentages. General levels of knowledge and perceptions towards flood disasters was also analyzed and presented using percentages and frequencies, followed by independent sample *t*-test. Lastly, responses regarding the willingness to report to work following a flood in addition to those related to the knowledge and competency concerning the flood scenario were analyzed using independent sample *t*-tests. Chi-square test was used for statistical testing and the significance was set to be less than 0.05.

## 3. Results

### 3.1. Demographic Characteristics

A total of 227 HCWs participated in this survey. The demographic characteristics can be found in Table 1. Males accounted for 77% of participants, with females accounting for 23%. Almost half of the total of participants (52%) belonged to the 26- to 34-year-old age group. Nearly one-third (29.1%) had 6 to 10 years of service, followed by 24.2% who had 2 to 5 years of service. A majority (94.7%) reported to work in the governmental sector. The highest numbers of participants were in Riyadh (74%) and married (65.6%). Meanwhile, 72.2% reported living with children. Approximately, half of the participants reported working in nursing divisions (52.4%), and nursing as a profession accounted for (40.1%).

### 3.2. Knowledge and Perceptions

In Table 2, an independent sample *t*-test was performed on gender for items related to participants’ perceptions towards their roles following a flood. Results demonstrated a significant statistical difference in the item related to familiarity with roles within the hospital’s operations following a flood (*p* < 0.01)—mean 3.44 ± 1.29 for males vs. 2.83 ± 1.26 for females. Due to a high percentage of those agreeing to the provided statements related to their roles following a flood, no significant differences were found. Furthermore, in perceptions related to the knowledge and competency concerning a presumed flood scenario, a statistically significant difference was found between males and females in terms of familiarity with the hospital’s standard operating procedure (*p* < 0.01)—mean 3.12 ± 1.33 for males vs. 2.57 ± 1.16 for females. Another significant difference was found related to male HCW confidence in managing a flood scenario (*p* < 0.05)—mean 3.32 ± 1.31 for males vs. 2.91 ± 1.26 for females. No other significant differences were detected (*p* > 0.05).

In Table 3, we report the results of independent sample *t*-tests to detect gender differences in the willingness to report to work after a flood; no statistically significant difference was reported (*p* > 0.05). However, when factors influencing the decision to report to work following a flood scenario was considered, a significant difference was found in females’ concerns for their families’ wellbeing (*p* < 0.01)—mean 4.89 ± 0.47 for females vs. 4.60 ± 1.04 for males. A statistically significant difference was found among females regarding concerns of houses being damaged as a consequence of the flood (*p* < 0.05)—mean 4.68 ± 0.78 for females vs. 4.34 ± 1.17 for males. With regards to professional commitment to care for the injured or ill victims, a statistical difference was found, with females having a higher commitment (*p* < 0.05)—mean 4.81 ± 0.56 for females versus 4.59 ± 1.03 for males.

Another statistically significant difference was found in Table 4 with regard to females’ perceptions towards the need for the development of guidelines for flood disasters and subsequent training in flood response (*p* < 0.05).

As reported in Appendix A, an average of three questions out of twelve were answered correctly for questions testing the knowledge and competency of the HCWs (range = 5.3% to 53.7%; mean = 25.4%; median = 21.6%). The highest percentages of correct answers per question were reported for responses regarding appropriate actions to be taken for a severely injured person, what is to be considered when an anxiety-stricken patient presents to the hospital, and the authority of issuing an evacuation of a department/unit (53.7%, 52%, and 38.3%, respectively). In contrast, the least correctly answered questions were reported for the questions regarding appropriate actions for a lightly injured casualty, the appropriate method of communications in the case of a shutdown, and the recommended treatment protocol for a casualty suffering from a crush injury (5.3%, 7.5%, and 14.1%, respectively). No significant associations were detected between the given answers and genders (*p* > 0.05).

Interpretation of the knowledge level score created by the authors can be found in Table 5. The answers provided by the participants indicate that almost all of them have low and moderate perceived knowledge levels (99.6%). Additionally, results showed that more than half of the participants scored a low knowledge level (60.8%) followed by a moderate knowledge level (38.8%). Only a single subject (0.4%) scored a high knowledge level, therefore it is not presented in the table. Chi-square test results demonstrated no associations between the level of knowledge and all the demographic information collected (*p* > 0.05). 

## 4. Discussion

This study assessed flood disaster preparedness among HCWs by measuring knowledge, attitudes, perceptions, and willingness to respond after a flood scenario. Nearly three-quarters of participants (73.2%) believed that hospitals are prepared to provide an effective response. Although many men had claimed to be familiar with their roles in the hospital’s operation following a flood, only 56% among all respondents actually felt that they were familiar with their roles following a disaster flood scenario. In parallel, women were reported to be less confident, but perhaps had more realistic views about flooding risks in Saudi Arabia. Though multi-agency collaboration has long been a good base for disaster management, good collaboration between organizations requires a common understanding of their emergency response responsibilities and organizational frameworks [22]. It is expected that if all stakeholders took part in a well-designed and practiced inter-agency all-hazards emergency response, HCWs at multiple hospitals would be more likely to have more faith in their expertise, skills, and competence [23]. Thus, through a case study in Saudi Arabia, it is suggested that the principle of collaboration and its implementation in disaster management should be revisited within the country.

In a cross-sectional survey conducted in the United States (US), results suggested that the majority of HCWs expected to be provided personal protective equipment as well as other measures to ensure hospital staff safety following a disaster [24,25]. Notably, 94% of the US study participants were confident about their hospitals’ abilities to respond effectively, with non-clinical staff found to be more confident when compared to clinical staff (OR 1.43, 95 % CI: 1.15–1.78) [25]. These findings are in contrast with results of a previous study, which reported high awareness among emergency physicians and nurses in SA [25]. In a local study, a large number of participants (85.7%) were confident in terms of their ability to handle disasters in a large tertiary hospital [25]. This discrepancy may be explained due to the nature of the study sample, which enrolled only emergency department staff [26,27].

When the flood scenario was proposed in this study, 45% of men believed that they were more familiar with the hospital’s standard operating procedures. Findings related to perceptions of knowledge and competency revealed that approximately 55% of all participants felt confident in the ability to treat flood victims. Concerns for personal safety, such as the hospital’s infrastructure being able to withstand a flood, was reported by 44.5% of participants. An Australian study also reported concerns among HCWs when asked about their personal preparedness. The study reported negative responses among non-emergency nursing staff and physiotherapists. Only a limited number of staff were capable of identifying their roles during a disaster response [28]. The findings in this study have identified a high number of HCWs (89%) who were willing to report to work immediately after a flood. The former percentage dropped slightly when the same surveyed staff were asked about expectations regarding their colleagues and peers (82.8%). Therefore, a low percentage of absenteeism could be expected. However, fear of losing jobs due to absence from work was a prevalent opinion among 55.1% of respondents. It is important to note that a higher percentage of respondents were working in nursing divisions (52.4%), a profession known to be dominated by expatriates in SA [29].

Conversely, an analysis of 2864 responses from an online survey of HCWs in the United States reported safety concerns as the most frequently cited barrier preventing workers from returning to work after an influenza pandemic, or any other disaster involving contagion or contamination [29]. The authors have acknowledged that studies concerned with workforce absenteeism during disasters is increasing, but in general remain an underrepresented issue in emergency planning efforts [30]. Quershi reported that 81% of 6628 HCWs from 47 healthcare facilities in New York City and the surrounding areas were willing to report to work during an environmental disaster [31]. Findings of the Quershi study were consistent with our results.

In this study, females were found to be more committed to reporting to work (*p* < 0.05) as well as more concerned for the wellbeing of their families (*p* < 0.01) and towards their houses sustaining damage due to a flood (*p* < 0.05). In a study by Cone and Cummings [29], data from 1711 respondents revealed that 87% were willing to work after mass casualty events, mainly in the case of natural disasters, but were less willing to return to work if a man-made catastrophe was suggested. While workers in such man-made incidents should not be endangered, disaster planners should consider that reassurance and assistance for HCWs may need to be handled differently. Several studies suggest that fears of one’s own safety, concern for the wellbeing of loved-ones, childcare, and other issues were linked to the failure of healthcare professionals to report for duty during crisis [31].

The need for developing flood disaster guidelines, accompanied with relevant training sessions of hospital staff, was a popular opinion for 90% of all participants––statistical significance was found among females (*p* < 0.05). The need for such guidance related to flood preparedness has been previously documented in other studies [32,33,34,35].

The hospital staff response was analyzed in terms of personal protective measures, case management/referral, and communication and competency skills listed in the protocols, policies, and procedures. Results of the flood scenario illustrated a surprising low percentage of correct answers, as stated previously. The scoring system from the checklist revealed that 99.6% of all HCWs demonstrated low to moderate levels of competency. Chi-square test results revealed no associations with the collected demographic information (*p* > 0.05). Our findings were consistent with a similar study in SA conducted solely on nurses that reported a lack of knowledge in regard to disaster and emergency preparedness [36].

To our best knowledge, this is the first study to assess flood disaster preparedness and the willingness to respond among healthcare workers in SA.

Findings of this study revealed multiple significant differences among the independent variables. Thus, it is believed that these findings can serve as a foundation for describing the current situation in the central region of SA.

## 5. Limitations

A limitation of this study is the potential for misclassification bias. This might be due to the adopted questionnaire being designed to measure disasters preparedness for an earthquake scenario. Nevertheless, key aspects of providing healthcare services during these responses are generally shared among natural disasters, and mainly affected by the management of assets, human resources, victims’ management and referral, mental health regulations, inter-agency collaboration, technology, information, communication, budget, and training management [37]. Another potential bias could be related to participants’ previous exposure and experiences with floods in the past. Geographic representation of the sample is considered to be another limitation, since participants represented mainly the central region of SA. While the current study demonstrated a lack of preparedness for flooding, further studies on all hazard emergencies preparedness might be of limited value due to the nature of disasters in the Saudi context [21].

## 6. Conclusions

The study demonstrated that levels of preparation for flood disaster management among healthcare providers in Saudi Arabia is inadequate for effective flood disaster responses. Our findings suggest that a majority of HCWs are confident of their hospitals’ preparedness to provide an effective response during flood disasters that is in line with the knowledge or theory side. Nevertheless, most of the participants (99.6%) demonstrated low and moderate levels of competency towards flood emergency preparedness due to the climate and geographical location of the kingdom. It is also estimated that a high percentage of HCWs are willing to report to work following a flood. Expected factors influencing the decision to report to work following a flood were concerns for their families’ wellbeing, as well as towards the security of their houses. Our study is consistent with the results in the literature, demonstrating the shortcomings related to preparedness and training for an actual flooding disaster [38]. While future training on general disaster response and preparedness and command center activities to enhance the collaborations between stakeholders seems to be crucial, additional areas of improvement needed for managing the impacts of future episodes of floods requires the development of national preparedness and training guidelines for hospitals in SA, including full-scale disaster exercises to measure the effectiveness of preparedness and response [39,40,41,42]. Directions for future research should focus on the differences in terms of preparedness among hospitals belonging to governmental sectors (MOH, MOE, MODA, MOI, and SANG) in order to tailor training programs according to regional- and/or hospital-specific contexts and needs, and should also direct additional focus on all hazard responses, command center operations, and communications [43,44,45,46,47].

Flooding today must be both appreciated and managed as multifactorial events [48,49,50,51,52]. Therefore, developing guidelines and standard operating procedures in addition to the introduction of educational interventions, such as training campaigns, and designing mobile solutions aimed to enhance the knowledge and awareness among HCWs is highly recommended [53,54,55,56,57,58,59,60,61,62,63,64,65,66,67]. It is essential to make a major shift toward improvement as far as the notion of flood disaster preparedness for healthcare providers is concerned. This study contributes to a fuller understanding of the needs of Saudi healthcare workers and may aid in their better planning for future flood disasters.

## Figures and Tables

**Table 1:** Demographic characteristics of the participants.

	Groups	Female (*n* = 53)	Male (*n* = 174)	All (*n* = 227)
Age	18–25 years	13 (24.5)	28 (16.1)	41 (18.1)
	26–34 years	24 (45.3)	94 (54)	118 (52)
	35–44 years	12 (22.6)	44 (25.3)	56 (24.7)
	45 and above	4 (7.5)	8 (4.6)	12 (5.3)
Length of Service	Less than 1	11 (20.8)	26 (14.9)	37 (16.3)
	2–5 years	19 (35.8)	36 (20.7)	55 (24.2)
	6–10 years	9 (17)	57 (32.8)	66 (29.1)
	11–15 years	8 (15.1)	40 (23)	48 (21.1)
	16–20 years	2 (3.8)	10 (5.7)	12 (5.3)
	20+ years	4 (7.5)	5 (2.9)	9 (4)
Scope of Hospital Practice	Private	1 (1.9)	9 (5.2)	10 (4.4)
	Government	52 (98.1)	163 (93.7)	215 (94.7)
Place of Residence	Riyadh	41 (77.4)	127 (73)	168 (74)
	Eastern Region	3 (5.7)	2 (1.1)	5 (2.2)
	Makkah	0 (0)	8 (4.6)	11 (2.2)
	Madinah	3 (5.7)	11 (6.3)	11 (4.8)
	Qassim	1 (1.9)	17 (9.8)	19 (8.4)
	Southern Region	3 (5.7)	2 (1.1)	3 (1.3)
	Northern Boarders	3 (0.6)	5 (2.9)	8 (3.5)
Family Status	Single	29 (54.7)	49 (28.2)	78 (34.4)
	Married	24 (45.3)	125 (71.8)	149 (65.6)
Number of Children	No Children	20 (37.7)	43 (24.7)	63 (27.8)
	1	11 (20.8)	37 (21.3)	48 (21.1)
	2	9 (17)	37 (21.3)	46 (20.3)
	3	5 (9.4)	29 (16.7)	34 (15)
	3+	8 (15.1)	28 (16.1)	36 (15.9)
Department	Nursing	40 (75.5)	79 (45.4)	119 (52.4)
	Physician	5 (9.4)	10 (5.7)	15 (6.6)
	Paramedic	0 (0)	15 (8.6)	18 (7.9)
	Pharmacy	3 (5.7)	15 (8.6)	15 (6.6)
	Other Clinical	2 (3.8)	41 (23.6)	43 (18.9)
	Support Services	0 (0)	9 (5.2)	9 (4)
	Fiscal and Administrative	3 (5.7)	5 (2.9)	8 (3.5)
Discipline	Nursing	26 (49.1)	65 (37.4)	91 (40.1)
	Physicians	15 (28.3)	18 (10.3)	33 (14.5)
	Pharmacy	3 (5.7)	14 (8)	17 (7.5)
	Administrative Professional/Secretary	2 (3.8)	11 (6.3)	13 (5.7)
	Other	7 (13.2)	66 (37.9)	73 (32.2)

Data are expressed as *n* (%).

**Table 2:** Knowledge and perceptions of participants.

		Knowledge and Perceptions as *n* (*%*)					*p*-Value (Independent Sample *t*-Test)
		Strongly Disagree	Disagree	Impartial	Agree	Strongly Agree	
Perceptions of the Role Following a Flood							
My role is vital to my organization’s effective management of a flood	Female	6 (11.3)	3 (5.7)	6 (11.3)	19 (35.8)	19 (35.8)	0.171
	Male	7 (4)	9 (5.2)	18 (10.3)	72 (41.4)	68 (39.1)	
	All *	13 (5.7)	12 (5.3)	24 (10.6)	91 (40.1)	87 (38.3)	
The hospital is prepared to provide an effective response to a flood	Female	4 (7.5)	1 (1.9)	12 (22.6)	26 (49.1)	10 (18.9)	0.478
	Male	8 (4.6)	22 (12.6)	14 (8)	79 (45.4)	51 (29.3)	
	All *	12 (5.3)	23 (10.1)	26 (11.5)	105 (46.3)	61 (26.9)	
I am familiar with my role in the hospital’s operation following a flood	Female	12 (22.6)	8 (15.1)	13 (24.5)	17 (32.1)	3 (5.7)	0.003
	Male	18 (10.3)	32 (18.4)	17 (9.8)	69 (39.7)	38 (21.8)	
	All *	30 (13.2)	40 (17.6)	30 (13.2)	86 (37.9)	41 (18.1)	
Perceptions of Knowledge and Competency Concerning a Flood Scenario							
I have sufficient knowledge concerning the treatment of flood victims	Female	8 (15.1)	14 (26.4)	8 (15.1)	19 (35.8)	4 (7.5)	0.068
	Male	24 (13.8)	28 (16.1)	20 (11.5)	73 (42)	29 (16.7)	
	All *	32 (14.1)	42 (18.5)	28 (12.3)	92 (40.5)	33 (14.5)	
I am familiar with the hospital’s standard operating procedure for floods	Female	12 (22.6)	15 (28.3)	11 (20.8)	14 (26.4)	1 (1.9)	0.007
	Male	29 (16.7)	36 (20.7)	16 (9.2)	71 (40.8)	22 (12.6)	
	All *	41 (18.1)	51 (22.5)	27 (11.9)	85 (37.4)	23 (10.1)	
I feel safe to stay at the hospital if a flood occurs	Female	9 (17)	10 (18.9)	7 (13.2)	19 (35.8)	8 (15.1)	0.468
	Male	26 (14.9)	24 (13.8)	25 (14.4)	73 (42)	26 (14.9)	
	All *	35 (15.4)	34 (15)	32 (14.1)	92 (40.5)	34 (15)	
I feel that I am competent as a caregiver to manage a flood	Female	12 (22.6)	7 (13.2)	9 (17)	24 (45.3)	1 (1.9)	0.043
	Male	22 (12.6)	31 (17.8)	23 (13.2)	65 (37.4)	33 (19)	
	All *	34 (15)	38 (16.7)	32 (14.1)	89 (39.2)	34 (15)	

Data are expressed as *n* (%); *: Both genders.

**Table 3:** Factors influencing decisions and willingness to report to work after floods.

	**Willingness to Report to Work Following a Flood—Opinion as *n* (%)**						***p*-Value** **(Independent Sample *t*-Test)**
		**No, I Don’t Believe I/They Will Show up**	**The Chances Are Low**	**I Can’t Decide**	**Yes, Almost Positive**	**Yes, without a Doubt**	
Will you report to work immediately after a flood?	Female	1 (1.9)	2 (3.8)	2 (3.8)	12 (22.6)	36 (67.9)	0.915
	Male	4 (2.3)	5 (2.9)	11 (6.3)	35 (20.1)	119 (68.4)	
	All ***	5 (2.2)	7 (3.1)	13 (5.7)	47 (20.7)	155 (68.3)	
In your opinion, will your colleagues report to work immediately after a flood?	Female	1 (1.9)	2 (3.8)	9 (17)	14 (26.4)	27 (50.9)	0.581
	Male	5 (2.9)	7 (4)	15 (8.6)	52 (29.9)	95 (54.6)	
	All ***	6 (2.6)	9 (4)	24 (10.6)	66 (29.1)	122 (53.7)	
	**Factors Influencing Decision to Report to Work Following a Flood—Opinion as *n* (%)**						***p*-value** **(Independent Sample *t*-test)**
		**Not at All**	**To a Small Extent**	**To an Undefinable Extent**	**To a Medium Extent**	**To a Large Extent**	
Concern for my family’s wellbeing	Female	0 (0)	1 (1.9)	0 (0)	0 (0)	49 (92.5)	0.006 **
	Male	9 (5.2)	5 (2.9)	3 (1.7)	12 (6.9)	145 (83.3)	
	All ***	9 (4)	6 (2.6)	3 (1.3)	15 (6.6)	194 (85.5)	
Concern that my house will be damaged in the flood	Female	0 (0)	3 (5.7)	1 (1.9)	6 (11.3)	43 (81.1)	0.018 *
	Male	8 (4.6)	14 (8)	9 (5.2)	22 (12.6)	121 (69.5)	
	All ***	8 (3.5)	17 (7.5)	10 (4.4)	28 (12.3)	164 (72.2)	
Professional commitment to care for the injured or ill	Female	0 (0)	1 (1.9)	1 (1.9)	5 (9.4)	46 (86.8)	0.038 *
	Male	6 (3.4)	8 (4.6)	6 (3.4)	12 (6.9)	142 (81.6)	
	All ***	6 (2.6)	9 (4)	7 (3.1)	17 (7.5)	188 (82.8)	
Fear of losing my place of employment due to my absence	Female	10 (18.9)	9 (17)	5 (9.4)	9 (17)	20 (37.7)	0.870
	Male	37 (21.3)	27 (15.5)	14 (8)	18 (10.3)	78 (44.8)	
	All ***	47 (20.7)	36 (15)	19 (8.4)	27 (11.9)	98 (43.2)	

***: Both genders; **: Significant at level 0.01; *: Significant at level 0.05.

**Table 4:** Perception towards guidelines development and training sessions.

	Developing a Guideline for Flood Disasters Accompanied with Training Sessions for Hospital Staff—Opinion as *n* (%)						*p*-Value (Independent Sample *t*-Test)
		Not at All	To a Small Extent	To an Undefinable Extent	To a Medium Extent	To a Large Extent	
Developing guidelines for flood disasters	Female	0 (0)	2 (3.8)	1 (1.9)	4 (7.5)	46 (86.8)	0.036 **
	Male	4 (2.3)	7 (4)	7 (4)	31 (17.8)	125 (71.8)	
	Total ***	4 (1.8)	9 (4)	8 (3.5)	35 (15.4)	171 (75.3)	
Attending training sessions on how to manage flood disasters	Female	0 (0)	3 (5.7)	0 (0)	2 (3.8)	48 (90.6)	0.021 *
	Male	7 (4)	10 (5.7)	5 (2.9)	20 (11.5)	132 (75.9)	
	Total ***	7 (3.1)	13 (5.7)	5 (2.2)	22 (9.7)	180 (79.3)	

*** Both genders; **: Significant at level 0.01; *: Significant at level 0.05.

**Table 5:** Level of knowledge and competency concerning a flood.

	Knowledge Level as *n* (%) ^§^			
	Groups	Low (*n* = 138)	Moderate (*n* = 88)	*p*-Value (Chi-Square Test)
Gender	Male	105 (46.5%)	68 (30.1)	0.485
	Female	33 (14.6%)	20 (8.8)	
	Total **	138 (61.5%)	88 (38.9)	
Age	18–25 years	22 (9.7%)	19 (8.4)	0.166
	26–34 years	72 (31.9%)	46 (20.4)	
	35–44 years	39 (17.3%)	16 (7.1)	
	45 and above	5 (2.2%)	7 (3.1)	
	Total **	138 (61.1%)	88 (38.9%)	
Length of Service	Less than 1	21(9.3%)	16 (7.1%)	0.531
	2–5	36 (15.9%)	19(8.4%)	
	6–10	38 (16.8%)	27 (11.9%)	
	11–15	33 (14.6%)	15 (6.6%)	
	16–20	5 (2.2%)	7 (3.1%)	
	20+ years	5 (2.2%)	4 (1.8%)	
	Total **	138 (61%)	88 (38.9%)	
Type of Hospital	Private	7 (3.1%)	3 (1.3%)	0.409
	Government	130 (58%)	84 (37.5%)	
	Total **	137 (60.6%)	87 (38.4%)	
Place of Residence	Riyadh	97 (43.3%)	70 (31.3%)	0.471
	Eastern Region	3 (1.3%)	2 (0.9%)	
	Makkah	8 (3.6%)	3 (1.3%)	
	Madinah	7 (3.1%)	4 (1.8%)	
	Qassim	12 (5.4%)	7 (3.1%)	
	Southern Region	3 (1.3%)	0 (0%)	
	Northern Boarders	7 (3.1%)	1 (0.4%)	
	Total **	137 (60.6%)	87 (38.4%)	
Family Status	Single	43 (19%)	34 (15%)	0.156
	Married	95 (42%)	54 (23.9%)	
	Total **	138 (61%)	88 (38.9%)	
Number of Children	0	38 (16.8%)	24 (10.6%)	0.690
	1	29 (12.8%)	19 (8.4%)	
	2	32 (14.2%)	14 (6.2%)	
	3	19 (8.4%)	15 (6.6%)	
	3+	20 (8.8%)	16 (7.1%)	
	Total **	138 (61%)	88 (38.9%)	
The Department of Workplace	Nursing	74 (32.7%)	44 (19.5%)	0.262
	Physician	10 (4.4%)	5 (2.2%)	
	Paramedic	5 (2.2%)	10 (4.4%)	
	Pharmacy	13 (5.8%)	5 (2.2%)	
	Other Clinical	24 (10.6%)	19 (8.4%)	
	Support Services	7 (3.1%)	2 (0.9%)	
	Fiscal and Administrative	5(2.2%)	3 (1.3%)	
	Total **	138 (61%)	88 (38.9%)	
Discipline	Nursing	51 (22.6%)	39 (17.3%)	0.148
	Physicians	25 (11.1%)	8 (3.5%)	
	Pharmacy	13 (5.8%)	4 (1.8%)	
	Other	49 (21.7%)	37 (16.4%)	
Total *	226 **	138 (61%)	88 (38.9%)	

^§^: The only single high knowledge score subject was excluded from the analysis of the Chi-square test to avoid statistical errors. *: Total number of participants with low and moderate knowledge scores. **: Both genders.

## Data Availability

Datasets used and analyzed during the current study are available from the corresponding author on reasonable request.

## Appendix A

**Table A1:** Questions and answers regarding knowledge and competency concerning a flood scenario.

Question	Correct Answers as *n* (%)				*p*-Value (Chi-Square Test)
	Categories	Female (*n* = 53)	Male (*n* = 174)	All (*n* = 227)	
What are the protection measures that should be provided for immobile patients during a flood?	Evacuate the patient with his/her bed to an external site outside the department ^a^	13 (24.5)	58 (33.3)	71 (31.3)	0.529
	Evacuate the patient with his/her bed to the departmental protected area	20 (37.7)	58 (33.3)	78 (34.4)	
	There is no way to protect immobile patients	1 (1.9)	5 (2.9)	6 (2.6)	
	Protect the patients in their beds by placing items under their beds to raise height of beds	6 (11.3)	10 (5.7)	16 (7)	
	I don’t know	13 (24.5)	43 (24.7)	56 (24.7)	
What are the personal protective actions the staff must implement during a flood?	Exit the department into the stairway all the way to the roof.	18 (34)	38 (21.8)	56 (24.7)	0.392
	Depart externally, outside of the hospital’s structure.	5 (9.4)	24 (13.8)	29 (12.8)	
	Avoid working alone and wear a coast guard-approved life jacket/vest ^a^	7 (13.2)	34 (19.5)	41 (18.1)	
	Wear a coast guard-approved life jacket/vest and avoid floodwater areas.	6 (11.3)	30 (17.2)	36 (15.9)	
	Dependent on the floor you are present in during the flood	15 (28.3)	41 (23.6)	56 (24.7)	
	I don’t know	2 (3.8)	7 (4)	9 (4)	
According to the standard operating procedure, what are the immediate actions to be implemented immediately following a flood?	Immediate evacuation of all patients from the department	14 (26.4)	53 (30.5)	67 (29.5)	0.128
	Identification of hospital’s departments that were damaged and provision of assistance as needed ^a^	5 (9.4)	40 (23)	45 (19.8)	
	Scout the area to locate casualties and damage	13 (24.5)	33 (19)	46 (20.3)	
	Concentrate staff and patients in the department and wait for instructions from the hospital management	4 (7.5)	13 (7.5)	17 (7.5)	
	I don’t know	17 (32.1)	35 (20.1)	52 (22.9)	
In case of potential damage to gas pipes and/or electricity supply infrastructure, what security measures should be implemented?	An immediate evacuation of the department	19 (35.8)	65 (37.4)	84 (37)	0.849
	The electricity, water, and gas supplies should be disconnected immediately	15 (28.3)	51 (29.3)	66 (29.1)	
	A substitute electricity source should be applied (a generator) ^a^	7 (13.2)	26 (14.9)	33 (14.5)	
	No action should be taken	0 (0)	2 (1.1)	2 (0.9)	
	I don’t know	12 (22.6)	30 (17.2)	42 (18.5)	
Who is authorized to issue an evacuation of a department/unit?	The hospital’s management solely	10 (18.9)	41 (23.6)	51 (22.5)	0.061
	The hospital’s management ^a^	15 (28.3)	72 (41.4)	87 (38.3)	
	The most senior member of the department	2 (3.8)	13 (7.5)	15 (.6)	
	The head of the department	11 (20.8)	18 (10.3)	29 (12.8)	
	I don’t know	15 (28.3)	30 (17.2)	45 (19.8)	
What medical registration procedures apply regarding admittance of patients to the hospital following a flood?	The routine medical registration will be continued	7 (13.2)	19 (10.9)	26 (11.5)	0.837
	Manual registration will be applied, and a list of casualties will be transferred ^a^	12 (22.6)	50 (28.7)	62 (27.3)	
	A small card will be utilized for every patient stating basic details and diagnosis	12 (22.6)	33 (19)	45 (19.8)	
	In an emergency situation, there is no time for an organized medical registration	6 (11.3)	25 (14.4)	31 (13.7)	
	I don’t know	16 (30.2)	47 (27)	63 (27.8)	
What is the recommended treatment protocol for a casualty suffering from a crush syndrome upon arrival at the hospital?	Amputation of the wounded limb	3 (5.7)	11 (6.3)	14 (6.2)	0.596
	Fasciotomy and extensive debridement of the necrotic muscle	9 (17)	34 (19.5)	43 (18.9)	
	Aggressive treatment with fluids and diuretics to prevent systemic complications ^a^	11 (20.8)	21 (12.1)	32 (14.1)	
	Immediate hemodialysis	1 (1.9)	2 (1.1)	3 (1.3)	
	I don’t know	29 (54.7)	106 (60.9)	135 (59.5)	
What is the appropriate action when a lightly injured casualty (for example, with limb wounds) presents to the hospital?	The patient should be directed to a designated site deployed outside the hospital area	12 (22.6)	47 (27)	59 (26)	0.925
	The patient should enter the hospital area and be provided with immediate treatment	22 (41.5)	65 (37.4)	87 (38.3)	
	The patient should be evacuated to a distant hospital for treatment ^a^	2 (3.8)	10 (5.7)	12 (5.3)	
	The patient should be directed to a designated site to be treated by a social worker/psychologist	3 (5.7)	8 (4.6)	11 (4.8)	
	I don’t know	14 (26.4)	44 (25.3)	58 (25.6)	
What is the appropriate action when a severely injured person (for example, suffering from crush syndrome or needing amputation) presents to the hospital?	The patient should be directed to one of the designated sites deployed outside the hospital area	7 (13.2)	25 (14.4)	32 (14.1)	0.532
	The patient should enter the hospital area and be provided with immediate treatment ^a^	32 (60.4)	90 (51.7)	122 (53.7)	
	The patient should be evacuated to a distant hospital for treatment	2 (3.8)	11 (6.3)	13 (5.7)	
	The patient should be directed to a designated site to be treated by a social worker/psychologist	0 (0)	9 (5.2)	9 (4)	
	I don’t know	12 (22.6)	38 (21.8)	50 (22)	
What is the appropriate action when an anxiety-stricken patient presents to the hospital?	The patient should be directed to one of the designated sites deployed outside the hospital area	6 (11.3)	25 (14.4)	31 (13.7)	0.687
	The patient should enter the hospital area and be provided with immediate treatment	2 (3.8)	15 (8.6)	17 (7.5)	
	The patient should be evacuated to a distant hospital for treatment	1 (1.9)	5 (2.9)	6 (2.6)	
	The patient should be directed to a designated site to be treated by a social worker/psychologist ^a^	31 (58.5)	87 (50)	118 (52)	
	I don’t know	13 (24.5)	42 (24.1)	55 (24.2)	
Following a flood, how will the control and communication inside the hospital be conducted?	No organized report mechanism can be implemented during an emergency	3 (5.7)	5 (2.9)	8 (3.5)	0.128
	There is need to report solely to the director of the emergency department	1 (1.9)	14 (8)	15 (6.6)	
	An emergency operation center will be created by the management ^a^	12 (22.6)	41 (23.6)	53 (23.3)	
	An emergency operation center will be created by the management and turned to only when needed	12 (22.6)	66 (37.9)	78 (34.4)	
	I don’t know	25 (47.2)	48 (27.6)	73 (32.2)	
Immediately following a flood, collapse of communication mechanisms may occur between the hospital and external institutions. Who should be reported to during this time?	The regional EMS (emergency medical services) center via ambulance drivers	25 (47.2)	90 (51.7)	115 (50.7)	0.339
	There is a need to wait for renewal of communication channels, and then a report should be submitted to the Ministry of Health	5 (9.4)	19 (10.9)	24 (10.6)	
	Media reporters (television, radio)	3 (5.7)	2 (1.1)	5 (2.2)	
	The police via local/field police teams ^a^	3 (5.7)	14 (8)	17 (7.5)	
	I don’t know	17 (32.1)	49 (28.2)	66 (29.1)	

Data are expressed as *n* (% of participants). ^a^ Correct answer according to published data.

## References

[1] A Global Facility for Disaster Reduction and Recovery (GFDRR) Saudi Arabia Page. https://www.gfdrr.org/en/saudi-arabia.

[2] World Health Organization Floods—Technical Hazard Sheet—Natural Disaster Profile Page. https://www.who.int/hac/techguidance/ems/floods/en/.

[3] Borunda A. Atmospheric Rivers Explained: Learn about the Weather Drenching California. https://www.nationalgeographic.com/environment/2019/03/atmospheric-river-flood-rain-california-explainer.

[4] Nunez C. Floods Explained. https://www.nationalgeographic.com/environment/natural-disasters/floods.

[5] Alderman K., Turner L.R., Tong S. (2012). Floods and human health: A systematic review. Environ. Int..

[6] Alraga S.M. (2017). An Investigation into Disaster Health Management in Saudi Arabia. J. Hosp. Med Manag..

[7] Tammar A., Abosuliman S.S., Rahaman K.R. (2020). Social Capital and Disaster Resilience Nexus: A Study of Flash Flood Recovery in Jeddah City. Sustainability..

[8] Shalhoub A.A., Khan A.A., Alaska Y.A. (2017). Evaluation of disaster preparedness for mass casualty incidents in private hospitals in Central Saudi Arabia. Saudi Med. J..

[9] Alamri Y.A. Emergency Management in Saudi Arabia: Past, Present and Future. https://training.fema.gov/hiedu/downloads/compemmgmtbookproject/comparative%20em%20book%20-%20em%20in%20saudi%20arabia.pdf.

[10] Tarawneh Q.Y., Chowdhury S. (2018). Trends of climate change in Saudi Arabia: Implications on water resources. Climate.

[11] Aljazeera At Least 12 People Killed as Floods Wreak Havoc in Saudi Arabia. https://www.aljazeera.com/news/2019/01/12-people-killed-floods-havoc-saudi-arabia-190130160250251.html.

[12] Al-Zahrani M., Al-Areeq A., Sharif H.O. (2017). Estimating urban flooding potential near the outlet of an arid catchment in Saudi Arabia. Geomat. Nat. Hazards Risk.

[13] Alshehri S.A., Rezgui Y., Li H. (2013). Public perception of the risk of disasters in a developing economy: The case of Saudi Arabia. Nat. Hazards.

[14] Abosuliman S.S., Kumar A., Alam F. (2013). Disaster preparedness and management in Saudi Arabia: An empirical investigation. Int. J. Soc. Hum. Sci. Eng..

[15] Al-Shareef A.S., Alsulimani L.K., Bojan H.M., Masri T.M., Grimes J.O., Molloy M.S., Ciottone G.R. (2017). Evaluation of hospitals’ disaster preparedness plans in the holy city of Makkah (Mecca): A cross-sectional observation study. Prehospital Disaster Med..

[16] Rattanakanlaya K., Sukonthasarn A., Wangsrikhun S., Chanprasit C. (2016). A survey of flood disaster preparedness among hospitals in the central region of Thailand. Australas. Emerg. Nurs. J..

[17] Shapira S., Friger M., Bar-Dayan Y., Aharonson-Daniel L. (2019). Healthcare workers’ willingness to respond following a disaster: A novel statistical approach toward data analysis. BMC Med. Educ..

[18] Shapira S., Aharonson-Daniel L., Bar-Dayan Y., Sykes D., Adini B. (2016). Knowledge, perceptions, attitudes and willingness to report to work in an earthquake: A pilot study comparing Canadian versus Israeli hospital nursing staff. Int. Emerg. Nurs..

[19] Brice J.H., Gregg D., Sawyer D., Cyr J.M. (2017). Survey of Hospital Employees’ Personal Preparedness and Willingness to Work Following a Disaster. South. Med. J..

[20] Al-Kibsi G., Woetzel J., Isherwood T., Khan J., Mischke J., Noura H. (2015). Saudi Arabia beyond oil: The investment and productivity transformation. McKinsey Glob. Inst..

[21] Virtual Snowball Sampling. https://www.ncbi.nlm.nih.gov/pmc/articles/PMC7691304/.

[22] Sulaiman N., She T.W., Fernando T., WeiChan S., Roslan A.F., Latib S.K. (2019). Multi-agency collaboration in flood disaster management in Sarawak, Malaysia. Int. J. Innov. Technol. Explor. Eng..

[23] Gebbie K.M., Weist E.M., McElligott J.E., Biesiadecki L.A., Gotsch A.R., Keck C.W., Ablah E. (2013). Implications of preparedness and response core competencies for public health. J. Public Health Manag. Pract..

[24] Mercer M.P., Ancock B., Levis J.T., Reyes V. (2014). Ready or not: Does household preparedness prevent absenteeism among emergency department staff during a disaster?. Am. J. Disaster Med..

[25] Ogedegbe C., Nyirenda T., DelMoro G., Yamin E., Feldman J. (2012). Health care workers and disaster preparedness: Barriers to and facilitators of willingness to respond. Int. J. Emerg. Med..

[26] Nofal A., Alfayyad I., Khan A., Al Aseri Z., Abu-Shaheen A. (2018). Knowledge, attitudes, and practices of emergency department staff towards disaster and emergency preparedness at tertiary health care hospital in central Saudi Arabia. Saudi Med. J..

[27] Raidla A., Darro K., Carlson T., Khorram-Manesh A., Berlin J., Carlström E. (2020). Outcomes of Establishing an Urgent Care Centre in the Same Location as an Emergency Department. Sustainability.

[28] Corrigan E., Samrasinghe I. (2012). Disaster preparedness in an Australian urban trauma center: Staff knowledge and perceptions. Prehospital Disaster Med..

[29] Alboliteeh M., Magarey J., Wiechula R. (2017). The profile of Saudi nursing workforce: A cross-sectional study. Nurs. Res. Pract..

[30] Garrett A.L., Park Y.S., Redlener I. (2009). Mitigating absenteeism in hospital workers during a pandemic. Disaster Med. Public..

[31] Qureshi K., Gershon R.R., Sherman M.F., Straub T., Gebbie E., McCollum M., Erwin M.J., Morse S.S. (2005). Health care workers’ ability and willingness to report to duty during catastrophic disasters. J. Urban Health..

[32] Cone D.C., Cummings B.A. (2006). Hospital disaster staffing: If you call, will they come?. Am. J. Disaster Med..

[33] Valdez C.D., Nichols T.W. (2013). Motivating healthcare workers to work during a crisis: A literature review. J. Manag. Policy Pract..

[34] Älgå A., Dang T.A., Saulnier D.D., Nguyen G.T., Von Schreeb J. (2018). Hope for the Best, Prepare for the Worst—An Assessment of Flood Preparedness at Primary Health Care Facilities in Central Vietnam. Int. J. Environ. Res. Public Health.

[35] Dube E., Mtapuri O., Matunhu J. (2018). Managing flood disasters on the built environment in the rural communities of Zimbabwe: Lessons learnt. Jàmbá: J. Disaster Risk Stud..

[36] Ibrahim F.A. (2014). Nurses knowledge, attitudes, practices and familiarity regarding disaster and emergency preparedness–Saudi Arabia. Am. J. Nurs. Sci..

[37] Pourhosseini S.S., Ardalan A., Mehrolhassani M.H. (2015). Key aspects of providing healthcare services in disaster response stage. Iran. J. Public Health.

[38] Khorram-Manesh A., Angthong C., Pangma A., Sulannakarn S., Burivong R., Jarayabhand R., Örtenwall P. (2014). Hospital evacuation; Learning from the past? Flooding of Bangkok 2011. J. Adv. Med. Med Res..

[39] Goniewicz K., Goniewicz M., Burkle F.M., Khorram-Manesh A. (2020). The Impact of Experience, Length of Service, and Workplace Preparedness in Physicians’ Readiness in the Response to Disasters. J. Clin. Med..

[40] Goniewicz K., Goniewicz M., Burkle F.M., Khorram-Manesh A. (2021). Cohort Research Analysis of Disaster Experience, Preparedness, and Competency-based Training among Nurses. PLoS ONE.

[41] Khorram-Manesh A., Ashkenazi M., Djalali A., Ingrassia P.L., Friedl T., von Armin G., Lupesco O., Kaptan K., Arculeo C., Hreckovski B. (2015). Education in disaster management and emergencies: Defining a new European course. Disaster Med. Public Health Prep..

[42] Hertelendy A.J., Goniewicz K., Khorram-Manesh A. (2020). The COVID-19 pandemic: How predictive analysis, artificial intelligence and GIS can be integrated into a clinical command system to improve disaster response and preparedness. Am. J. Emerg. Med..

[43] Goniewicz K., Magiera M., Burkle F., Goniewicz M. (2020). Prospective Study on the Potential Use of Satellite Data for Disaster Prevention, Preparedness, and Mitigation in Poland. Prehospital Disaster Med..

[44] Goniewicz K., Magiera M., Rucińska D., Pawłowski W., Burkle F.M., Hertelendy A.J., Goniewicz M. (2020). Geographic information system technology: Review of the challenges for its establishment as a major asset for disaster and emergency management in Poland. Disaster Med. Public Health Prep..

[45] O’Shea T., Bates P., Neal J. (2020). Testing the impact of direct and indirect flood warnings on population behaviour using an agent-based model. Nat. Hazards Earth Syst. Sci..

[46] Ab Aziz N.F., Akashah F.W., Aziz A.A. (2019). Conceptual framework for risk communication between emergency response team and management team at healthcare facilities: A Malaysian perspective. Int. J. Disaster Risk Reduct..

[47] Casado M.R., Leinster P. (2020). Towards more effective strategies to reduce property level flood risk: Standardising the use of Unmanned Aerial Vehicles. J. Water Supply Res. Technol. Aqua.

[48] Goniewicz K., Burkle F. (2019). Analysis of the Potential of IT System Support in Early Warning Systems: Mitigating Flood Risk in Poland. Prehospital Disaster Med..

[49] Raikes J., Smith T.F., Jacobson C., Baldwin C. (2019). Pre-disaster planning and preparedness for floods and droughts: A systematic review. Int. J. Disaster Risk Reduct..

[50] Nagendra N.P., Narayanamurthy G., Moser R. (2020). Management of humanitarian relief operations using satellite big data analytics: The case of Kerala floods. Ann. Oper. Res..

[51] Bailey K., Williford K., Cawley P., Wain M.J., Lehman-Huskamp K. (2020). Operational Communications: Adapting a Hospital Daily Check-In (DCI) for Expanded Use During Emergency Events. Disaster Med. Public Health Prep..

[52] Hassan W.H., Jidin A.Z., Aziz S.A.C., Rahim N. (2019). Flood disaster indicator of water level monitoring system. Int. J. Electr. Comput. Eng..

[53] Burkle F.M., Bradt D.A., Ryan B.J. (2020). Global Public Health Database Support to Population-Based Management of Pandemics and Global Public Health Crises, Part I: The Concept. Prehospital Disaster Med..

[54] Burkle F.M., Bradt D., Green J., Ryan B. (2020). Global Public Health Database Support to Population-Based Management of Pandemics and Global Public Health Crises, Part II: The Database. Prehospital Disaster Med..

[55] Al-Wathinani A., Hertelendy A.J., Alhurishi S., Mobrad A., Alhazmi R., Altuwaijri M., Alanazi M., Alotaibi R., Goniewicz K. (2021). Increased Emergency Calls during the COVID-19 Pandemic in Saudi Arabia: A National Retrospective Study. Healthcare.

[56] Salmoral G., Casado M.R., Muthusamy M., Butler D., Menon P.P., Leinster P. (2020). Guidelines for the Use of Unmanned Aerial Systems in Flood Emergency Response. Water.

[57] Gupta K. (2020). Challenges in developing urban flood resilience in India. Philos. Trans. R. Soc. A.

[58] Fang Z., Wang Y., Peng L., Hong H. (2020). Predicting flood susceptibility using long short-term memory (LSTM) neural network model. J. Hydrol..

[59] Goniewicz K., Goniewicz M. (2020). Disaster Preparedness and Professional Competence Among Healthcare Providers: Pilot Study Results. Sustainability.

[60] Pour S.H., Abd Wahab A.K., Shahid S., Dewan A. (2020). Low impact development techniques to mitigate the impacts of climate-change-induced urban floods: Current trends, issues and challenges. Sustain. Cities Soc..

[61] Goniewicz K., Burkle F.M. (2019). Disaster early warning systems: The potential role and limitations of emerging text and data messaging mitigation capabilities. Disaster Med. Public Health Preparedness.

[62] Parnell A., Goniewicz K., Khorram-Manesh A., Burkle F.M., Al-Wathinani A., Hertelendy A.J. (2020). COVID-19 a health reform catalyst? Analyzing single-payer options in the US: Considering economic values, recent proposals, and existing models from abroad. J. Hosp. Adm..

[63] Almoradie A., De Brito M.M., Evers M., Bossa A., Lumor M., Norman C., Yira Y., Hounkpe J. (2020). Current flood risk management practices in Ghana: Gaps and opportunities for improving resilience. J. Flood Risk Manag..

[64] Khorram-Manesh A., Burkle F.M., Phattharapornjaroen P., Marzaleh M.A., Al Sultan M., Mäntysaari M., Carlstrom E., Goniewicz K., Santamaria E., Comandante J.D. (2020). The Development of Swedish Military Healthcare System: Part II-Re-evaluating the Military and Civilian Healthcare Systems in Crises Through a Dialogue and Study Among Practitioners. Military Med..

[65] Khorram-Manesh A., Carlström E., Hertelendy A.J., Goniewicz K., Casady C.B., Burkle F.M. (2020). Does the prosperity of a country play a role in COVID-19 outcomes?. Disaster Med. Public Health Prep..

[66] Goniewicz K., Goniewicz M., Włoszczak-Szubzda A., Burkle F.M., Hertelendy A., Al-Wathinani A., Molloy M.S., Khorram-Manesh A. (2021). The importance of pre-training gap analyses and the identification of competencies and skill requirements of medical personnel for mass casualty incidents and disaster training. BMC Public Health.

[67] Barendrecht M.H., Sairam N., Cumiskey L., Metin A., Holz F., Priest S., Kreibich H. (2020). Needed: A systems approach to improve flood risk mitigation through private precautionary measures. Water Secur..

